# Lactate metabolism-related genes to predict the clinical outcome and molecular characteristics of endometrial cancer

**DOI:** 10.1186/s12885-023-10934-y

**Published:** 2023-05-31

**Authors:** Rui Shi, Haojia Li, Sitian Wei, Zhicheng Yu, Jun Zhang, Qi Zhang, Ting Zhou, Yuwei Yao, Qian Zhang, Tangansu Zhang, Hongbo Wang

**Affiliations:** Department of Gynecology and Obstetrics, Union Hospital, Tongji Medical College, Huazhong University of Science and Technology, Wuhan, Hubei People’s Republic of China

**Keywords:** Endometrial cancer, Lactate metabolism-related gene, TIMM50, Proliferation, Migration

## Abstract

**Background:**

Metabolic reprogramming is one of hallmarks of cancer progression and is of great importance for the tumor microenvironment (TME). As an abundant metabolite, lactate has been found to play a critical role in cancer development and immunosuppression of TME. However, the potential role of lactate metabolism-related genes in endometrial cancer (EC) remains obscure.

**Methods:**

RNA sequencing data and clinical information of EC were obtained from The Cancer Genome Atlas (TCGA) database. Lactate metabolism-related genes (LMRGs) WERE from Molecular Signature Database v7.4 and then compared the candidate genes from TCGA to obtain final genes. Univariate analysis and Least Absolute Shrinkage and Selection Operator (LASSO) Cox regression were performed to screen prognostic genes. A lactate metabolism-related risk profile was constructed using multivariate Cox regression analysis. The signature was validated by time-dependent ROC curve analysis and Kaplan-Meier analysis. The relationship between the risk score and age, grade, stage, tumor microenvironmental characteristics, and drug sensitivity was as well explored by correlation analyses. Gene ontology (GO) enrichment analysis and Kyoto Encyclopedia of Genes and Genomes (KEGG) pathway functional analysis between the high and low-risk groups were performed. CCK8, EdU, and clone formation assays were applied to detect the proliferation ability of EC cells, Transwell assay was performed to detect the migration ability of EC cells, and intracellular lactate and glucose content was used to asses lactate metabolism.

**Results:**

We constructed a risk signature based on 18 LMRGs. Kaplan-Meier curves confirmed that the high-risk group had poorer prognosis compared to the low-risk group. A nomogram was then constructed to predict the probability of EC survival. We also performed GO enrichment analysis and KEGG pathway functional analysis between the high and low-risk groups, and the outcome revealed that the features were significantly associated with energy metabolism. There was a significant correspondence between LMRGs and tumor mutational load, checkpoints and immune cell infiltration. C1, C2, and C4 were the most infiltrated in the high-risk group. The high-risk group showed increased dendritic cell activation, while the low-risk group showed increased plasma cells and Treg cells. Drug sensitivity analysis showed LMRGs risk was more resistant to Scr kinase inhibitors. We further proved that one of the lactate metabolism related genes, TIMM50 could promote EC cell proliferation, migration and lactate metabolism.

**Conclusion:**

In conclusion, we have established an effective prognostic signature based on LMRG expression patterns, which may greatly facilitate the assessment of prognosis, molecular features and treatment modalities in EC patients and may be useful in the future translation to clinical applications. TIMM50 was identified as a novel molecule that mediates lactate metabolism in vitro and in vivo, maybe a promising target for EC prognosis.

**Supplementary Information:**

The online version contains supplementary material available at 10.1186/s12885-023-10934-y.

## Introduction

EC is one of the most common cancers among women, with 417,000 new diagnoses worldwide in 2020 [1]. A woman’s lifetime risk of developing EC is approximately 3%, and the median age at diagnosis was 61 years. The overall incidence has increased by 132% over the past 3 decades, suggesting that risk remain prevalent globally, particularly in obesity and aging population [2]. The clinical features of EC usually present as post-menopausal bleeding, but only 5–10% of women with post-menopausal bleeding are associated with the potential for endometrial malignancy [3]. The diagnosis of EC patients is dependent on the histological examination of endometrial tissue samples, however, this invasive test is only available for patients with endometrial lesions or endometrial thickening demonstrated by transvaginal ultrasound scanning [4]. The prognosis of advanced or specific pathological types of EC is unsatisfied, so identifying novel molecular markers and prognostic indicators of EC is essential to improve treatment and reduce the burden of disease.

Metabolic reprogramming is an essential hallmark of cancer, and metabolic reprogramming is relied by cancer cells for their ability to grow and proliferate within a nutrient-poor TME [5]. Metabolic reprogramming in cancer primarily includes glycolysis, increased glutamine consumption, and increased fatty acid synthesis [6]. Otto Warburg [7] discovered the different metabolic characteristics of cancer cells in the 1920s. Despite sufficient oxygen, tumor cells over-intake glucose and preferentially produce lactic acid, whereas normal cells normally utilize oxidative phosphorylation. This “Warburg effect” or aerobic glycolysis has been demonstrated in many tumor types [8]. Worenine targets HIF-1α to inhibit the Warburg effect in colorectal cancer cells thereby suppressing tumor cell growth, proliferation and cell cycle progression [9]; Melatonin inhibits lung cancer progression by stimulating the SIRT3/PDH axis to reverse the Warburg effect [10]; PGC1α suppresses HCC metastasis by regulating PPARγ-dependent WNT/β-catenin/PDK1 axis to inhibit aerobic glycolysis [11];The phosphorylation of AMPKα1 by PIM2 in EC leads to a decrease in AMPKα1 kinase activity, which in turn promotes aerobic glycolysis and tumor growth [12]. However, the mechanism of action of lactate, a major product of glycolysis, in EC is poorly understood.

The end product of glycolysis, lactate, has been found to have an important role in energy regulation, wound healing, ischemic tissue damage, memory formation, and cancer growth and metastasis [13, 14]. The concentration of lactate in tumor tissues is significantly higher than that in normal tissues, and the increased serum concentration of lactate is associated with metastasis and rapid growth of tumors [15]. A growing number of studies suggest that proton-coupled lactate efflux from cancer cells or stromal cells plays a critical role in maintaining an acidic phenotype and increasing tumor progression by regulating TME, including cell invasion, angiogenesis, survival signaling, metastasis development, and escape from immunosurveillance [16]. Lactate induces M2 polarization in tumor-associated macrophages and secretes CCL17 to promote pituitary adenoma invasion [17]. PD-L1 expression on neutrophils is induced by tumor-derived lactate via the MCT1/NF-κB/COX-2 pathway, thereby enhancing resistance to levatinib in hepatocellular carcinoma [18]. However, research on the mechanism of lactate in EC and tumor microenvironment have not been investigated.

In our study, we analyzed RNA-seq data and clinical data in EC to screen for LMRGs differentially expressed in EC tissues and normal tissues, and constructed a prognostic signature for LMRGs. Next the correlation between LMRG and immune-infiltrating cells in TME was analyzed, and drug sensitivity analysis was performed. We validated in vitro that TIMM50 enhanced EC cell proliferation, migration and lactate levels, and promoted tumor growth in vivo. The results showed that LMRGS was of high value in predicting the prognosis of EC.

## Method

### Data Acquisition

Fragments per kilobase transcript(FPKM) data for EC were downloaded from the TCGA data portal(https://portal.gdc.cancer.gov/), which includes 23 normal and 552 tumor samples. The somatic mutation profile, and corresponding clinical information of EC were as well obtained from the TCGA data portal. Data processing was performed using Perl software to generate a matrix of all differential genes in EC. Data analysis was performed with R software (version 4.2.1) and R Bioconductor packages.

### Lactate-related genes screening

By searching with the keywords “lactate” the Molecular Signatures database v7.4 (MSigDB; https://www.gsea-msigdb.org/gsea/msigdb) [19], and 5 lactate-related pathways (GOBP_LACTATE_METABOLIC_PROCESS、HP_INCREASED_SERUM_LACTATE、HP_LACTIC_ACIDOSIS、HP_LACTICACIDURIA、HP_SEVERE_LACTIC_ACIDOSIS) were obtained, and 289 lactate metabolism-related genes were integrated by eliminating the duplicated genes. The mRNA expression matrix and LMRGs-related genes were analyzed by the limma package in R, and the expression matrix of LMRGs-related genes was extracted. 123 genes with a fold change (FC) > 1.5 and false discovery rate (FDR) < 0.05 were defined as differentially expressed LMRGs between tumor samples and adjacent tissues.

### Development of a lactate-based signature for prognosis and somatic mutation

We further filtered these 123 lactate phenotype-associated genes for prognostic markers. Using univariate Cox regression analysis, we identified 18 prognostic markers were used as lactate-related markers with p-values < 0.05. The coefficients of each marker were determined by Cox regression models. Tumor mutation burden analysis was conducted on the 18 genes screened using the “maftools” package in R. To avoid overfitting, we further performed LASSO Cox regression (1000 iterations) with the “glmnet” package and the “survival” [20–22]. Following the screening of the LASSO regressions, the selected LMRGs were used to establish the LMRGS via multivariate Cox regression analysis.

### Establishment of LMRGs prognostic signature

The LMRGS score was calculated as the following formula: LMRGs score = expression level of gene1 × coefficient of gene1 + expression level of gene2 × coefficient of gene2 +. . + expression level of genen ×coefficient of gene_n_ [23]. The risk score of each patient was predicted by using the ‘predict’ function included in the survival R package. Two subgroups according to the median LMRGs score of EC patients were classified, including the LMRGs-high and the LMRGs-low groups. To evaluate the accuracy of grouping, we performed principal component analysis for all differential genes, LMRGs and riskGenes. To assess the prognostic value of LMRGs, we performed Kaplan-Meier (KM) survival analysis to compare overall survival (OS) and Progression free survival (PFS) between the two groups of LMRGs. To explore the impact of LMRGS on EC progression, we elucidated the relationship between LMRGS and clinicopathological factors, including age, stage, pathological grade and risk score. To estimate the accuracy of the constructed models, multivariate independent prognostic analyses were performed and ROC curves for the above clinical characteristics were plotted using the “timeROC” package, “survival” package and, “survminer” package.

### Predictive nomogram establision

To investigate the prognostic effect of lactate fraction, the clinical characteristics were integrated in multivariate Cox regression analysis. The prognostic nomogram to predict the 1-year, 3-year, and 5-year OS of EC patients in the TCGA dataset were constructed [24]. Calibration curves were employed to assess the consistency between predicted survival and actual survival. The ROC curves over time were employed to evaluate the specificity and sensitivity of the model.

### Immune microenvironment evaluation

Thorsson etc. [25] have developed a new global immune classification of solid tumours based on the transcriptomic profiles of over 10,000 patients from all 33 non-haematological The TCGA cancer types in 2019. Six distinct immune subtypes (ISs) were identified, including C1 (wound healing), C2 (IFN-gamma dominant), C3 (inflammatory), C4 (lymphocyte depleted), C5 (immunological quiet), and C6 (TGF-beta dominant). The relationship between risk score and immunophenotyping were calculated using the “ggpubr” package. To detect immune cell infiltration in EC patients, the R package CIBERSORT (CIBERSORT, script v1.03) were utilized to estimate the abundance of 22 immune cell types based on gene expression data. The immune-associated signatures from previous studies were referenced and calculated their scores using genomic variance analysis (GSVA).

### Drug sensitivity calculation

To evaluate the clinical application of LMRGs in EC therapeutics, the IC50 of chemotherapeutic agents commonly used in the EC dataset TCGA project was calculated using the algorithm developed by Geeleher et al. and the corresponding R package ‘pRRophetic‘ [26, 27]. This algorithm permits the user to predict clinical chemotherapy response by using only baseline tumor gene expression data, which is achieved by taking cell lines from the Cancer Genome Project’s Gene expression and drug sensitivity data from cell lines in the Cancer Genome Project to build statistical models. Differences in IC50s for common antineoplastic drugs between the high- and low-risk groups of LMRGs were compared using the Wilcoxon signed rank test.

### Functional Enrichment

For LMRGs, GO and KEGG pathway enrichment analyses (showing the top 20) were performed with the R package clusterProfiler and were plotted as box plots and bubble plots, respectively. GO analyses consisted of biological processes (BP), cellular components (CC), and molecular functions (MF). The KEGG database has been developed as a computer model of biological information systems represented in terms of molecular interaction and reaction networks [28]. We have got permission to use the KEGG software from the Kanehisa laboratory. GO and KEGG pathways were identified as significantly enriched with a P value < 0.05. For high and low-risk groups, the activity of signature pathways was analyzed using GSVA.

### Quantitative real-time polymerase chain reaction validation(qRT-PCR)

Total RNA from cells was extracted by using TRizol (#9108, RNAiso Plus, Takara, Japan) in accordance with the instructions. The cDNA was obtained with PrimeScriptRT Reagent Kit (#RR037A, Takara, Japan). qRT-PCR was conducted by Biosystem StepOne Plus PCR System (ABI) and Real-Time PCR Kit (Takara, Japan). The expression levels of RNA were calculated by the 2-ΔΔCT method using the expression of GAPDH as an internal reference. qRT-PCR with sense and antisense primers were as follows: GAPDH 5′-GGCAGAGATGATGACCCTTTT-3′ and 5′-AGATCCCTCCAAAATCAAGTGG-3′; TIMM50: 5′- ACTGTGCACGAGGTTGGCGA-3′ and 5′-GTCCACCGGGTTGTTTCCAAAG-3′.

### Cell culture

EC cell lines consisted of HEC-1B, HEC-1 A, Ishikawa, RL-952, and KLE, which were purchased from the American Type Culture Collection (ATCC; Manassas, VA). Ishikawa, HEC-1B, RL-952, and KLE cells were cultured with DMEM/F12 medium (#11,330,032, Gibco), HEC-1 A cells were cultured with McCoy’s 5 A medium (#16,600,082, Gibco), complemented with 10% fetal bovine serum (#10,099,141, Gibco) and 1% streptomycin and penicillin (#PYG0016, Bosterbio, USA) in a 37 °C, 5% CO2 incubator.

### Transfection

The shRNA of TIMM50 was purchased from GenChem(Shanghai, China). EC cells were seeded in six-well plates and transfected with TIMM50 shRNA and control shRNA when the fusion level reached 60%-70%. TIMM50-sh1: 5’-CCGGACAUACAAAUAUUUUCAA-3’; TIMM50-sh2: 5’-GACACCAUGUAAAGGAUAUUU-3’; TIMM50-sh3: 5’-CCUCAAGACCAUUGCACUGAA-3’; TIMM50-Negative control: 5’-UUCUCCGAACGUGUCACGU-3’.

### EdU assay

Cells(1*10^4 cells / well)were plated in 96-well plates and incubated overnight for EdU (5-ethynyl-2′ deoxyuridine) assay. Edu assay was performed using Edu kit (#C0071S, Beyotime, shanghai), according to the instructions. The EdU solution was prepared as a 1:1000 concentration of medium and added to the pre-prepared 96-well plates with 100 ul per well, which were incubated at 37 °C for 2 h and fixed in 4% paraformaldehyde for 15 min. The fixed cells were incubated with 50ul of reaction solution for 30 min sheltered from light, and then the nuclei were stained for 10 min by using DAPI. Cell proliferation was imaged by utilization of a 20x fluorescence microscope.

### Cell counting Kit-8 assays

Cell Counting Kit-8 Assay (#34,302, CCK8, Bimake, USA) was applied to detect cell proliferation. Cells (4 × 10^3/well) were cultured in 96-well plates and CCK8 solution and cell culture medium mixture(1:9) were added to 96-well plates, shielded from light, and incubated at 37℃ for 1 h. The absorbance was measured at 450 nm at four time points: 24 h, 48 h, 72 and 96 h as instructed. The absorbance was measured by using an automated microplate reader (BioTek, VT, USA).

### Migration assays

The 8 μm pore size chambers were used for migration experiments. 200 µl of serum-free cell suspension containing Ishikawa cells and HEC-1B cells (1 × 10^4/well) was added above the chambers, and 600 µl of medium containing 20% fetal bovine serum was added below the chambers. After 24 h of incubation, the chambers were removed, fixed with 4% paraformaldehyde for 10 min, stained with crystal violet, and photographed with a 10x microscope.

### western blot (WB) analysis

Total cellular protein was extracted utilizing RIPA lysate, and protein quantification was performed by BCA protein assay. Electrophoresis was performed using a 10% PAGE gel, followed by transfer to a 0.2 μm PVDF membrane on a Trans-Blot Turbo transfer system. After 1.5 h of skim milk closure and overnight incubation with primary antibody at 4 °C, the membranes were washed with TBST. The membranes were processed with diluted secondary antibodies for 1.5 h at room temperature. Proteins were visualized with ECL detection reagents and ChemiDoc imaging system. Since the molecular weight of TIMM50 protein was 40 kDa, the membrane was cut at around 40 kDa and incubated with antibody.

### Immunohistochemistry (IHC)

Sections were dissociated with xylene, rehydrated with ethanol, and antigen retrieval was performed in a microwave oven (121 °C, 15 min). Endogenous peroxidase activity was obstructed with 5% H_2_O_2_. Sections were then incubated in 10% normal mouse serum to block nonspecific binding of the antibody and incubated overnight at 4 °C with primary antibody TIMM50 (1:200, ABclonal, Wuhan, China). Sections were subsequently incubated with secondary antibodies for 2 h at room temperature, immersed in 0.1% diaminobenzidine tetrahydrochloride solution for 5 min, and counterstained with hematoxylin.

### HE staining

Dewaxing, 10 min each for dewaxed xylene I and II, coverslips prepared in advance. Overlay water, 100% (I, II), 90%, 80%, 70% alcohol for 5 min each, tap water rinse for 5 min × 3. Hematoxylin staining for 5 min, rinsed with running water. 5% acetic acid differentiation for 1 min, rinse with running water, add acetic acid by pipette drops, cloth can be covered with the tissue on the slide, the color became lighter and blue after differentiation. Eosin staining for 1 min. Dehydration: 70%, 80%, 90%, 100% alcohol for 10 s each, xylene for 1 min, can be dried naturally in the fume hood and then sealed piece, about 5 min or so. Drop on neutral gum, seal the film.

### Lactate and glucose measurement

For cells in wall culture, the cells were scraped off with a cell scraper and the culture medium was centrifuged at 1000 rpm for 10 min at room temperature, and the supernatant was discarded and the cell sediment was left. Add 0.5ml of PBS to the cell precipitate and mix well, suspend the cells in PBS, transfer the cell suspension to a glass homogenization tube (2ml glass homogenization tube) with a pipette, place the glass homogenization tube in ice water mixture and homogenize manually for 3 min, then take the broken cell suspension for determination of lactate and glucose content.

### Tumor xenograft assay

Female BALB/c-nu nude mice (4–5 weeks old) (Charles River, China) were housed in a standard pathogen-free environment laboratory. Mice were randomly divided into two groups, sh-NC and sh-TIMM50. Treated HEC-1B (1 × 10^6^) cells were suspended in 100 µl serum-free MEM medium and subsequently injected subcutaneously into the right shoulder nail of mice. The subcutaneous tumor size was measured weekly, and after 28 days, the mice were treated with cervical dislocation and the subcutaneous graft tumors were removed, and the weight, length and width of the tumors were measured. Tumor volume = (length × width^2^)/2. Tumor samples were partially embedded in paraffin for histopathological analysis.

### Statistical analysis

Quantitative analysis was performed using appropriate statistical methods. Student’s t-test or Wilcoxon signrank test was applied for comparisons between the two groups and one-way ANOVA for comparisons between more than two groups, depending on whether the samples conformed to parametric tests [29, 30]. KaplanMeier survival rates were compared across subgroups using the log-rank test. The Spearman correlation method was employed to test the correlation between the two groups of numerical variables. Cox proportional hazards models were applied to analyze hazard ratios (HR) with 95% confidence intervals (95% CI). A value of P less than 0.05 was considered significant. The statistical analyses were performed in R or Graphpad.

## Result

### Identification of lactate metabolism related differentially expressed genes of EC in the Cancer Genome Atla

**Fig. 1:**
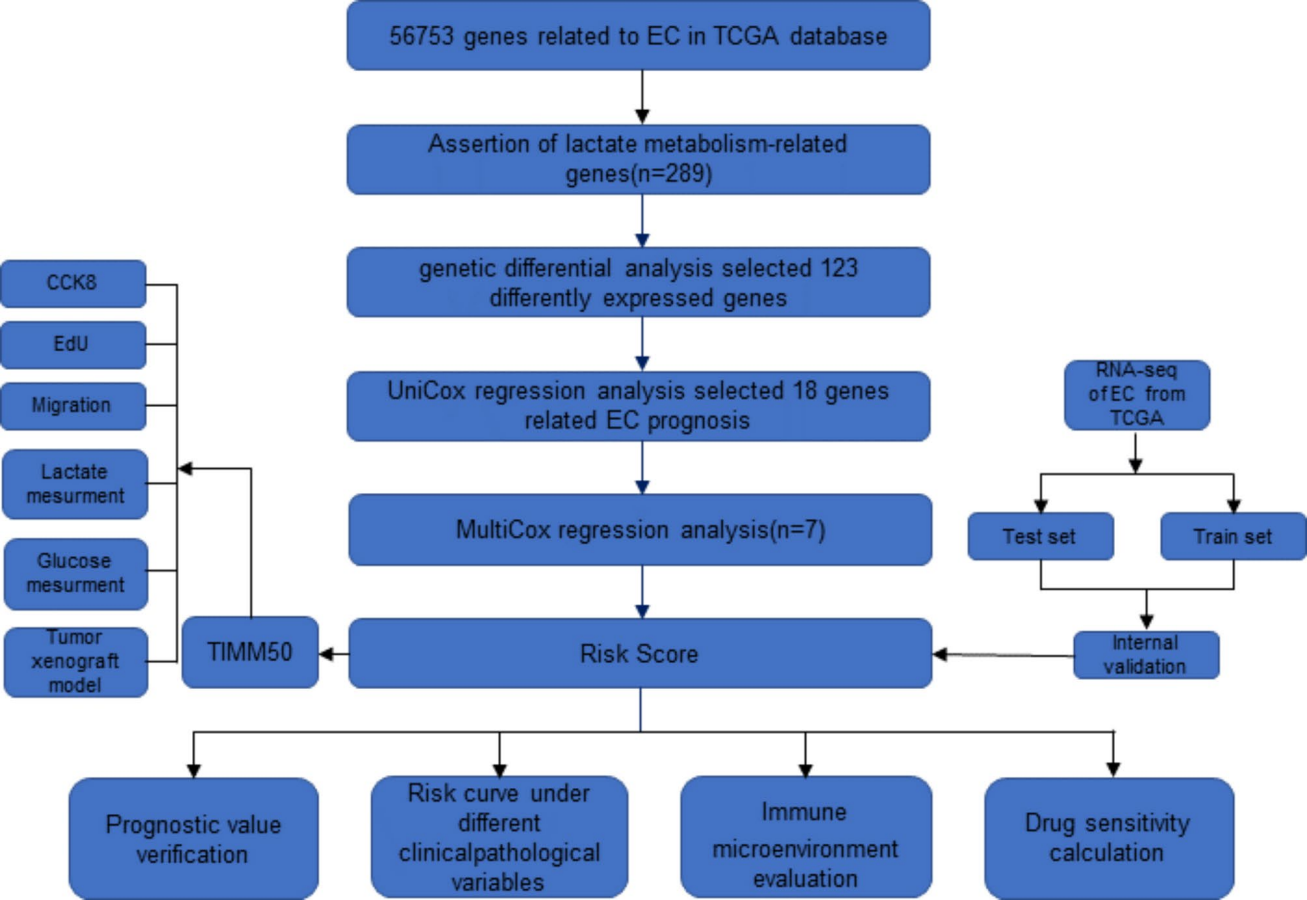
Flow diagram of the study

**Fig. 2:**
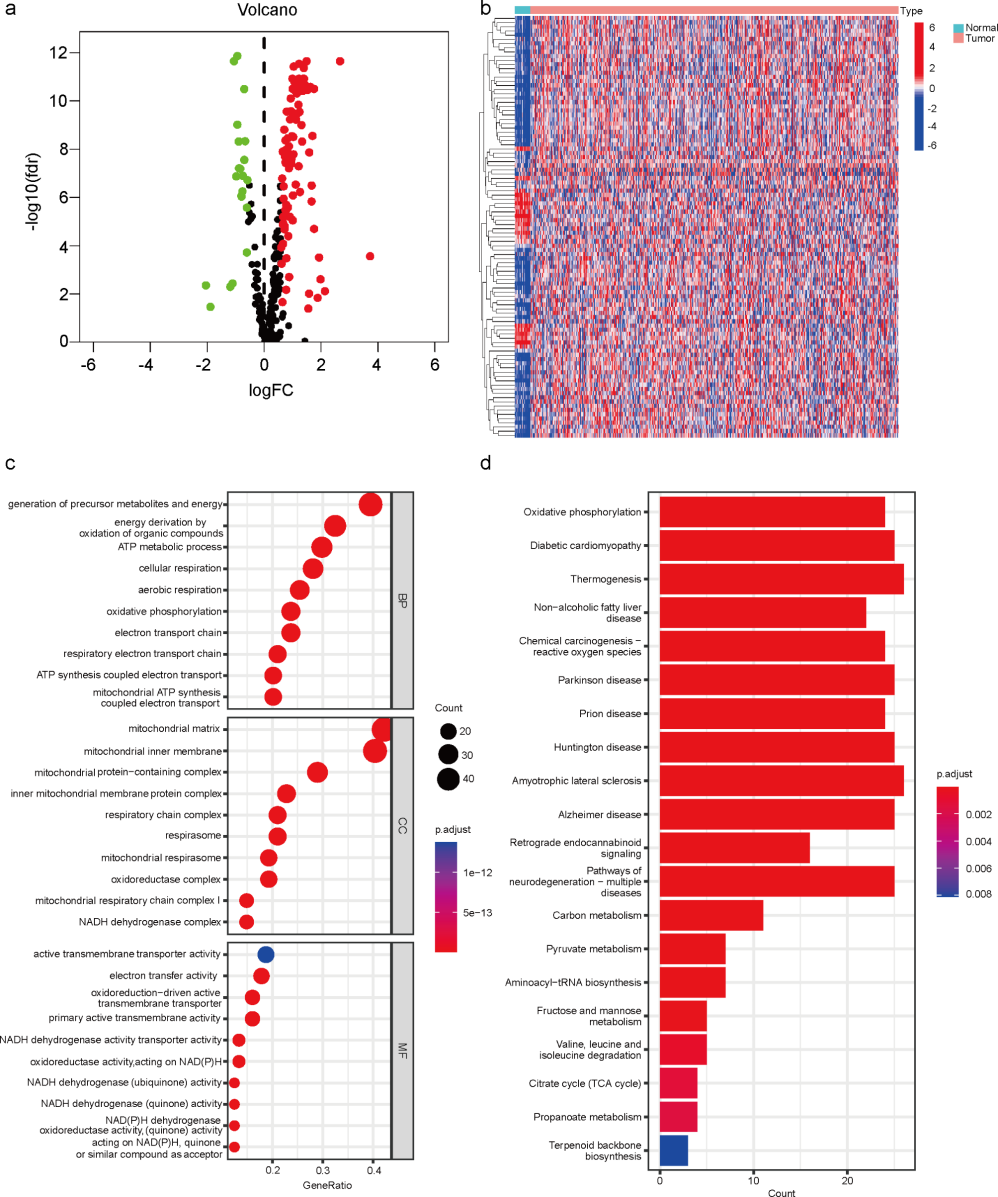
Identification of lactate metabolism related differentially expressed genes of EC in The Cancer Genome Atla. **a** Volcano plot demonstrating the down- and up-regulated LMRGs. **b** Heat map displaying the expression levels of LMRGs in each sample. **c** GO enrichment analysis. **d** KEGG pathway enrichment analysis

RNA-sequencing and clinical data of 552 EC and 23 normal endometrium tissues were downloaded from the TCGA database. To understand the metabolic characteristics of lactate in EC, 289 lactate genes from 5 lactate metabolic pathways were integrated in MSigDB. The workflow of the lactate metabolism-related gene signature analysis is demonstrated in Fig. 1. Based on RNA-seq profiling of the TCGA EC cohort, 122 were identified differentially expressed between EC tissue and normal endometrial tissue. Heat map and volcano map demonstrated 19 genes were low expression, whereas 103 genes high expression (Fig. 2a-b). To further explore the function of differentially expressed lactate metabolism-related genes (LMRGs) in EC, GO functional enrichment analysis and KEGG signaling pathway analysis on 122 lactate genes were performed. As shown in Fig. 2c, the BP is involved in generation of precursor metabolites and energy, energy derivation by oxidation of organic compounds, ATP metabolic process, cellular respiration, aerobic respiration, electron transport chain. The CC were mainly participated in the mitochondrial matrix, mitochondrial inner membrane, mitochondrial protein-containing complex, respiratory chain complex, NADH dehydrogenase complex, etc. The MF were mainly focused on electron transfer activity, oxidoreduction-driven active transmembrane transporter, NADH dehydrogenase activity and transporter activity. KEGG enrichment analysis was focused on Oxidative phosphorylation, Diabetic cardiomyopathy, and Thermogenesis (Fig. 2d).

### Screening of prognostic genes for LMRGs and the mutation landscape of EC

**Fig. 3:**
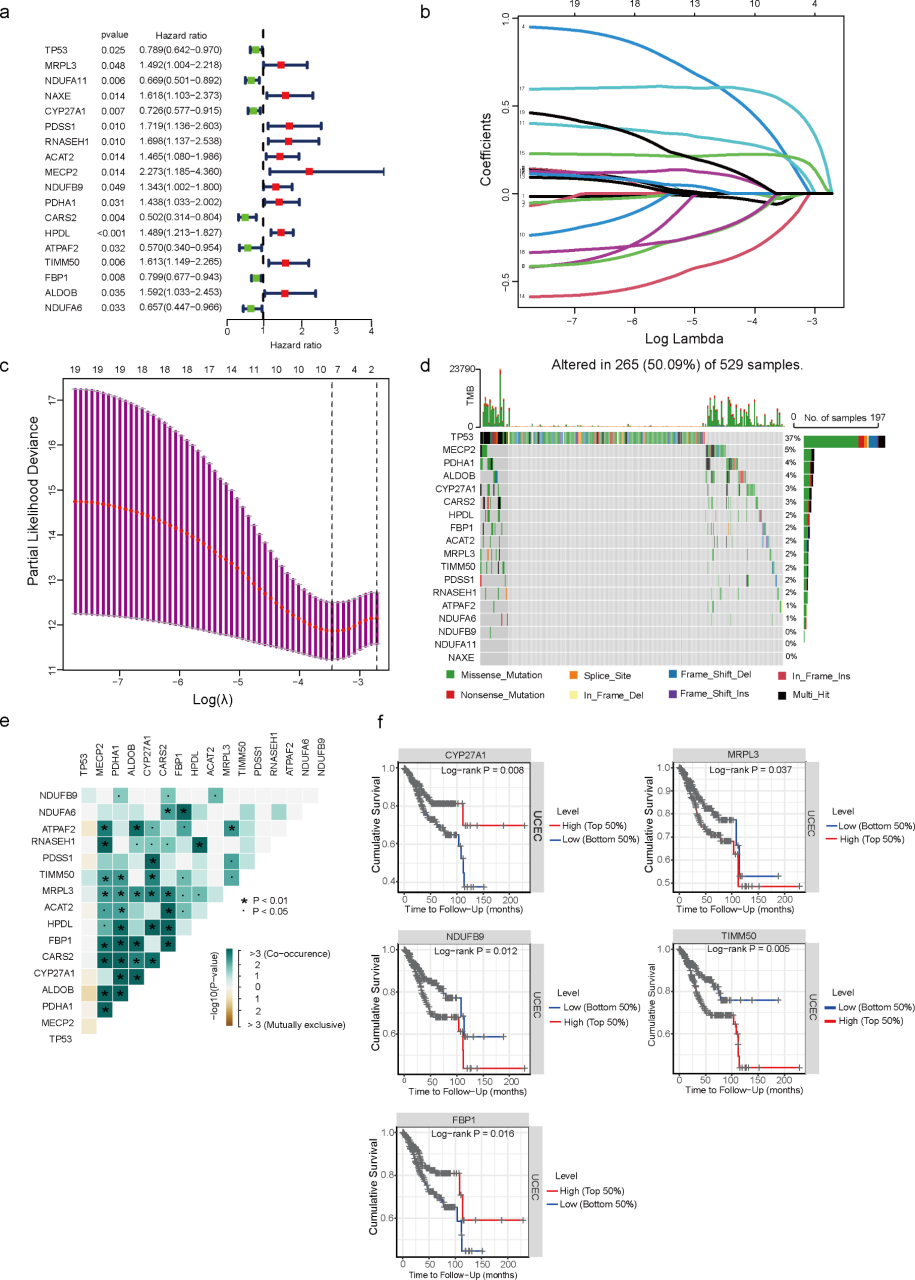
Screening of prognostic genes for LMRGs and the mutation landscape of EC. **a** Univariate Cox regression analysis to screen 18 prognosis-related LMRGs. **b** LASSO coefficient curves of prognosis-related LMRGs. **d, e** Statistical analysis of mutation frequencies of lactate-related genes in EC. **f** Prediction of correlation between lactate-related genes and prognosis of EC patients by Timer

To confirm the association between LMRGs and prognosis, univariate COX regression analysis was performed and 18 genes were identified to be associated with EC prognosis, including TP53, MRPL3, NDUFA11, NAXE, CYP27A1, PDSS1, RNASEH1, ACAT2, MECP2, NDUFB9, PDHA1, CARS2, HPDL, ATPAF2, TIMM50, FBP1, ALDOB, NDUFA6(Fig. 3a). After LASSO regression analysis and multifactorial COX regression analysis(Table 1), only 7 genes were eventually acquired (Fig. 3b-c). Tumor mutation load of EC patients were downloaded from the TCGA database and the 18 genes were screened univariately. The results indicated that TP53 was the most mutated gene (Fig. 3d-e). To further validate the screened genes, the online database TIMER was used for prediction and CYP27A1, MRPL3, NDUFB9, TIMM50, and FBP1 were associate with EC patient prognosis. Of these, CYP27A1 and FBP1 were positively associated with prognosis of EC patients, while the opposite was observed for MRPL3, NDUFB9, and TIMM50 (Fig. 3f).

**Table 1:** Multifactorial COX regression analysis of LMRGs

id	coef	HR	HR.95 L	HR.95 H	pvalue
NAXE	0.29402	1.341811	0.90023	1.999998	0.148787
CYP27A1	-0.21527	0.806325	0.638348	1.018506	0.0709
ACAT2	0.369831	1.447489	1.030769	2.032682	0.032769
CARS2	-0.36432	0.694667	0.427241	1.129483	0.141831
TIMM50	0.483174	1.621212	1.172321	2.241986	0.003487
ALDOB	0.510096	1.665451	1.120277	2.47593	0.01169
NDUFA6	-0.58768	0.555615	0.370907	0.832306	0.004369

### Establishment and validation of LMRGs prognostic signature for patients with EC

**Fig. 4:**
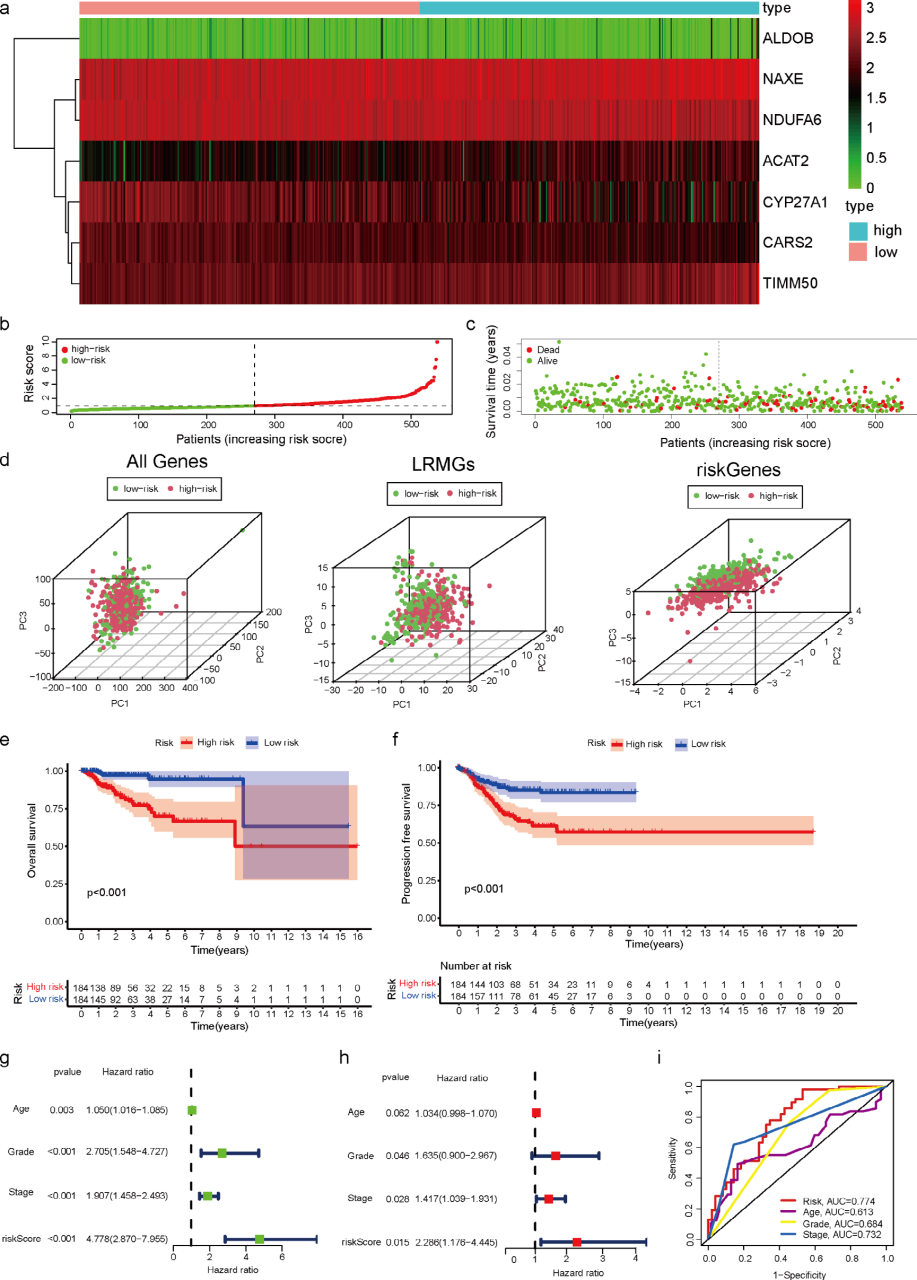
Establishment and validation of LMRGs prognostic signature for patients with EC. **a** Multivariate Cox regression analysis of LMRGs. **b, c** Risk score distribution and survival status of EC samples based on the risk score. **d** The PCA, based on all genes, lactate-related genes, and riskgenes, indicate that tumor samples and adjacent tissues are wellseparated by first principal component (PC1) and second principal component (PC2). **e, f** The Kaplan?Meier survival curves of OS and PFS according to high-and low-risk groups in patients with EC. **g, h** The univariate Cox regression and multivariate analysis of age, grade, stage, and risk score for OS. **i** ROC indicated the predictive accuracy of Riskscore was superior to other clinical parameters

To construct a reliable prognostic signature for EC patients, EC patients were categorized into a high-risk group and a low-risk groups based on the median risk score. Heat map analysis (Fig. 4a) shown seven genes were differentially expressed in the high- and low-risk groups, which were ALDOB, NAXE, NDUFA6, ACAT2, CYP27A1, CARS2, and TIMM50. Patients were categorized into two groups based on the optimal cutoff value for the risk of LMRGs for diagnostic accuracy (Fig. 4b). More mortality events were observed in the high-risk group, suggesting that the increased risk of LMRGs reflected an unfavorable prognosis for EC patients (Fig. 4c). Principal component analysis (PCA) at different levels was employed to verify whether the risk states among different influencing factors were relatively independent. PCA demonstrated that samples with two risk scores were divided into two independent groups (Fig. 4d). Kaplan–Meier survival curves analyses showed that the high-risk subgroup had a shorter OS and PFS than the low-risk groups (Fig. 4e-f). To further investigate whether the prognostic signature could be an independent predictor of survival in EC, univariate and multivariate Cox regression analyses were performed. Univariate Cox regression analysis revealed that risk score was an independent predictor of poor OS in EC patients (HR = 4.778, 95% CI: 2.870–7.955). The results of multivariate COX regression analysis were the same (Fig. 4g-h). Remarkably, the predictive capability of these traditional clinical parameters (age, grade, and stage) was significantly lower than that of the LMGRs risk score. ROC curves of clinically relevant factors were conducted to assess the performance of the risk prediction model, which described excellent predictive capability. The AUCs for risk, age, grading and staging were 0.774, 0.613, 0.684 and 0.732, respectively (Fig. 4i). To prove the stability of the model, 503 EC patients (entire set) were randomly allocated to the train set (n = 352) and test set (n = 151) by seven-to-three ratio. The distribution of risk score (Supplement Fig. 1a), the survival status (Supplement Fig. 1b), the survival outcome(Supplement Fig. 1c-d), univariate and multivariate Cox regression analyses and the ROC curve (Supplement Fig. 1e-f) of EC patients between two groups of the train set and test set were constructed, respectively. All results in the two sets show no difference. The risk curves and scatter plots for the train and test sets implied mortality was positively related to the risk score in two sets. Kaplan–Meier survival analysis indicated that patients in the low-risk groups demonstrated better OS and PFS than patients in the high-risk groups in train and test sets. Univariate and multivariate Cox regression analyses and ROC curves were performed. Remarkably, the predictive capability of these traditional clinical parameters (age, grade, and stage) was significantly lower than that of the LMGRs risk score of train and test sets. ROC curves, assessing the accuracy of this risk model in two sets, indicated that the LMGRs model is reliable and precise. Risk score could be used as an effective prognostic marker in EC.

### Construction of the nomogram

**Fig. 5:**
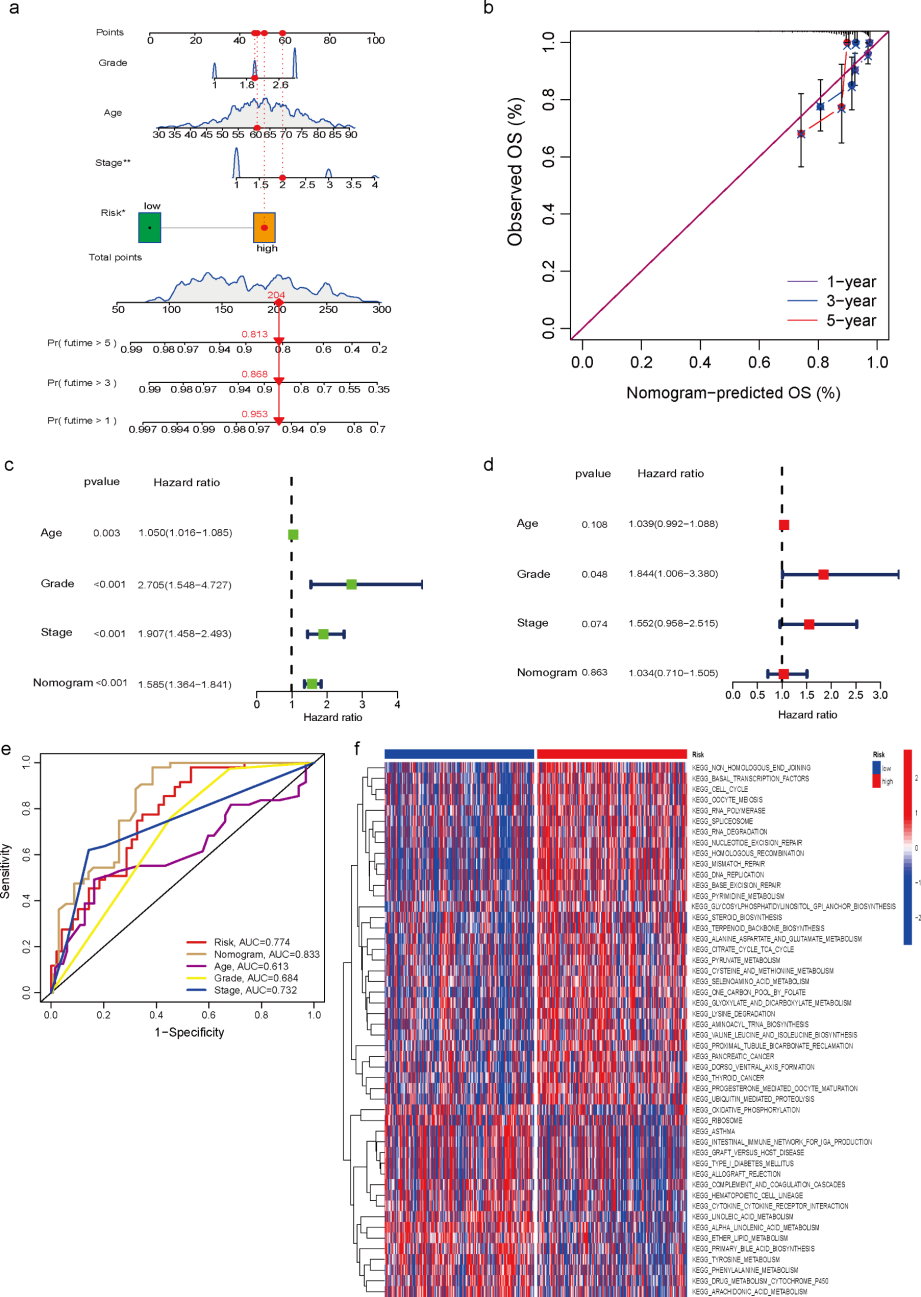
Construction of the nomogram. **a** The prognostic nomogram was plotted to predict 1-, 3-, and 5-year survival probability of EC patients. **b** The calibration plot was performed to evaluate the prediction consistency of OS. **c, d** The univariate Cox regression and multivariate analysis of age, grade, stage, and nomogram for OS. **e** ROC curves of age, grade, stage, risk, and the nomogram. **f** KEGG pathway enrichment analysis of different risk scores

To accurately predict the probability of OS, a nomogram integrating the LMRGs risk score and other clinicopathological features, including age, stage, and grad was constructed to assess survival at 1, 3 and 5 years. The results suggested that the OS of EC patients decreased along with increasing time (Fig. 5a). Moreover, the calibration curve was designed to assess the predictive accuracy of 1-, 3-, and 5-year survival rates. Interestingly, the predicted risk was similar to the actual risk, indicating the reliability of our nomogram (Fig. 5b). To further determine whether nomogram could be used as an independent prognostic indicator for EC patients, univariate and multivariate Cox regression analyses were performed. The outcomes showed that in the univariate Cox analysis, age, stage, grade, and nomogram were all significantly related to the probability of OS. However, in multivariate COX regression analysis, grade was as well an independent risk factor for prognosis in EC patients (Fig. 5c-d). ROC curves were plotted for nomogram versus clinical characteristics, and the results demonstrated that nomogram (AUC = 0.833) exhibited more favorable clinical predictive accuracy than any single factor (Risk, AUC = 0.774; age, AUC = 0.613; grade, AUC = 0.684; stage, AUC = 0.732) (Fig. 5e). Eventually, KEGG signaling pathway analysis was performed for the high- and low-risk groups, which were mainly enriched in Basal Transcription factors, Cell Cycle, Glycosylphosphatidylinositol gpi anchor biosynthesis, alanine aspartate and glutamate metabolism, citrate cycle TCA cycle, pyruvate metabolism, cysteine and methionine metabolism, et al. (Fig. 5f).

### Comparison of immune activity and the sensitivity to anticancer drugs between patients with different risk scores

**Fig. 6:**
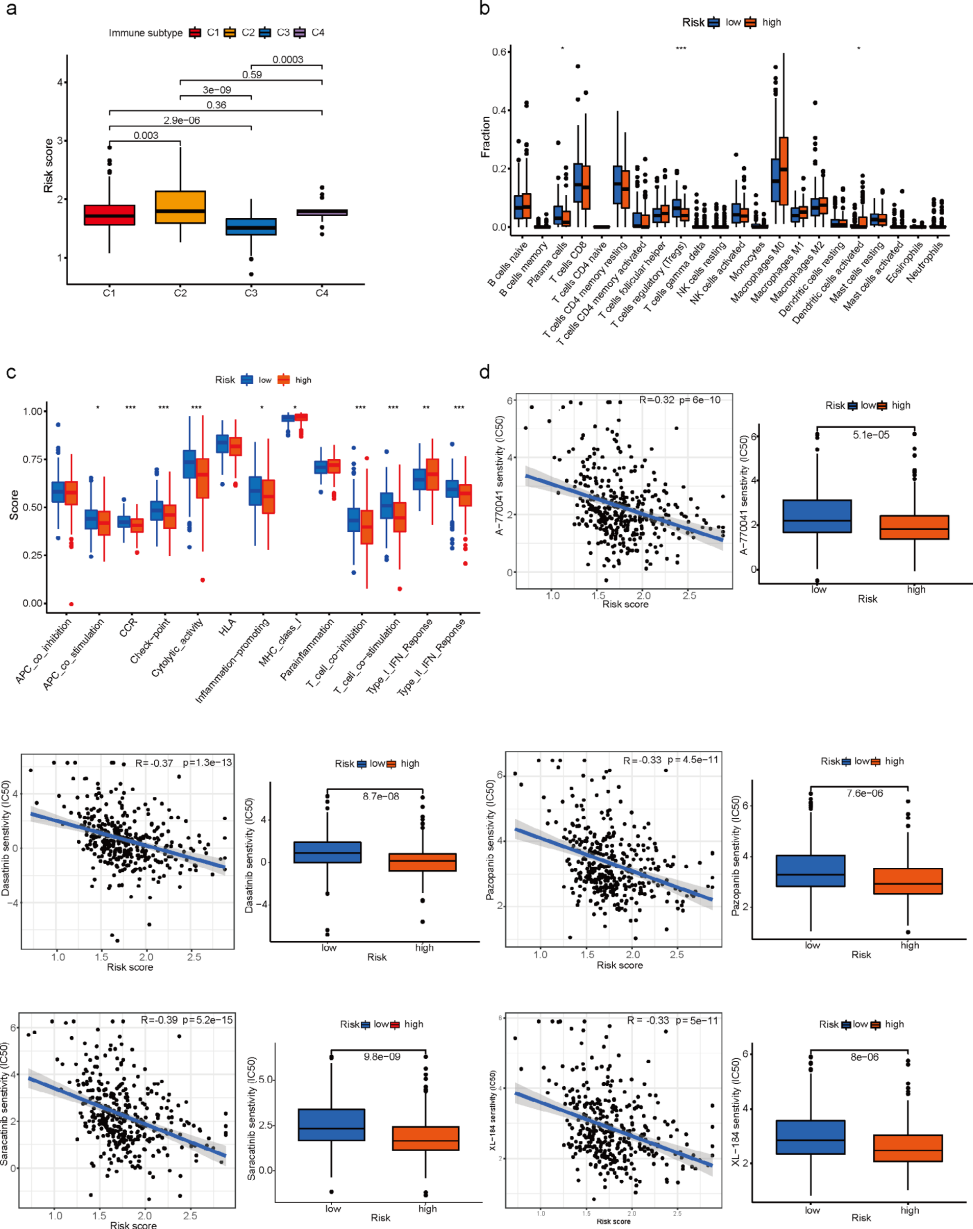
Comparison of immune activity and the sensitivity to anticancer drugs between patients with different risk scores. **a** The analysis of the correlation between immunotyping and risk scores. **b** Box plot for comparing the infiltration percentage of 22 immune cells between LMRGS-low and LMRGS-high groups. **c** Box plot for comparing the immune signatures of the two risk groups, as calculated by GSVA. **d** Estimated drug sensitivity in patients with high and low LMRGs risk.

The analysis of the correlation between immunotyping and risk scores revealed that C3 was the most infiltrated in the low-risk groups and significantly different from C1, C2 and C4(Fig. 6a). To thoroughly analyze the immune microenvironment, CIBERSORT was used to calculate the degree of infiltration of 22 immune cell types. As shown in Fig. 6b, infiltration of Plasma cells, T cells regulatory (Tregs), and Dendritic cells activated were significantly different in the high and low-risk groups of EC patients. To know more about the activation of immune-related functions in EC, the correlation analysis of immune function and risk scores was conducted. The results(Fig. 6c) showed that APC co-stimulation, CCR, Check-point, Cytolytic activity, Inflammation-promoting, MHC class I, T cell co-inhibition, T cell co-stimulation and Type II IFN Response were more active in the low-risk groups compared to the high-risk group, while Type I IFN Response showed the opposite trend. Sensitivity to common anticancer drugs was compared between the high- and low-risk groups to determine potential treatment modalities for EC. The results showed that IC50s for Scr protein kinase inhibitors were higher in low-risk patients, suggesting that low LMRGs risk was more resistant to Scr kinase inhibitors compared to high-risk (Fig. 6d). These drugs might be the future guidance for the treatment of patients at the high-risk score of EC.

### Knockdown of TIMM50 suppressed proliferation, migration and lactate synthesis of EC cell

**Fig. 7:**
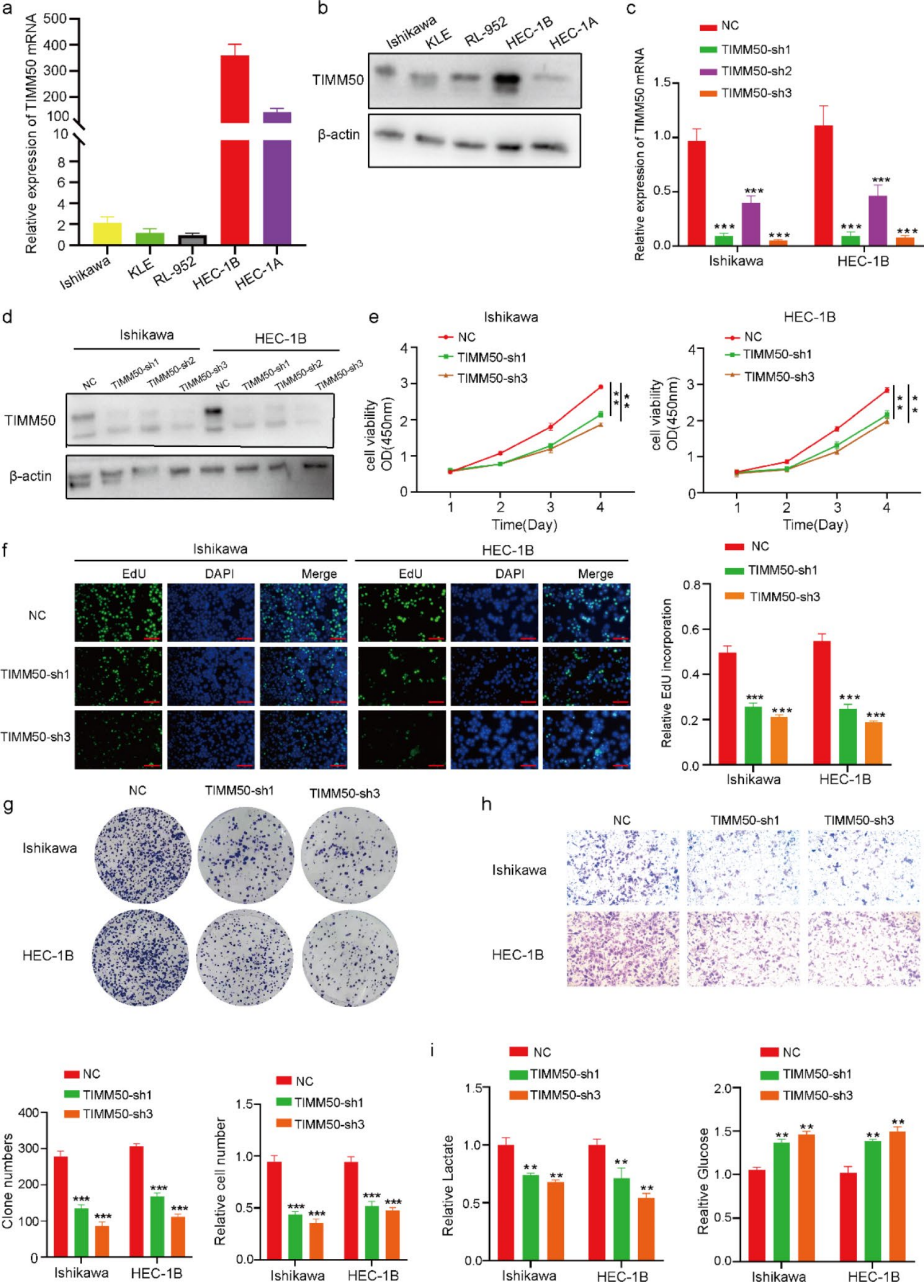
Knockdown of TIMM50 suppressed EC cell proliferation, migration, and lactate synthesis. **a, b** qRT-PCR and Western blot analysis of TIMM50 expression in EC cells(The samples derive from the same experiment and that gels/blots were processed in parallel). **c, d** qRT-PCR and Western blot analysis of TIMM50 expression in Ishikawa cells and HEC-1B cells after transfection of the shRNA vector(The samples derive from the same experiment and that gels/blots were processed in parallel). **e, f, g** CCK-8, EdU binding and colony formation assays were performed to evaluate the proliferation of Ishikawa cells and HEC-1B cells. **h** Transwell assays were conducted to assess the migration of Ishikawa cells and HEB-1C cells. **i** Lactate and glucose assays were used to evaluate the lactate metabolism of Ishikawa cells and HEB-1C cells. Scale bar, 100 μm. n = 3, *P < 0.05, **P < 0.01, ***P < 0.001

To explore the exact roles of TIMM50, qRT-PCR and Western Blot were performed to detect the expression of TIMM50 in different cell lines of EC in terms of RNA and protein level, respectively (Fig. 7a-b). TIMM50-si was transfected into Ishikawa and HEC-1B cells to verify its knockdown efficiency (Fig. 7c-d). CCK-8 assay showed that TIMM50 suppression repressed the viability of Ishikawa and HEB-1 C cells (Fig. 7e). EdU assay suggested that the inhibition of TIMM50 enhanced the apoptosis of Ishikawa and HEC-1B cells (Fig. 7f). The colony formation assay indicated that TIMM50 interference significantly restrained the colony formation ability of Ishikawa and HEC-1B cells (Fig. 7g). Transwell assay revealed that TIMM50 silencing repressed the invasion ability of Ishikawa and HEC-1B cells (Fig. 7h). Lactate and glucose assay assays (Fig. 7i) suggested that lactate synthesis was reduced in Ishikawa and HEB-1 C cells by silencing TIMM50, while glucose content showed the opposite trend. Hence, the ability of proliferation, migration and lactate synthesis of EC cells could be promoted by TIMM50 in vitro.

### TIMM50 enhance tumour growth in vivo

**Fig. 8:**
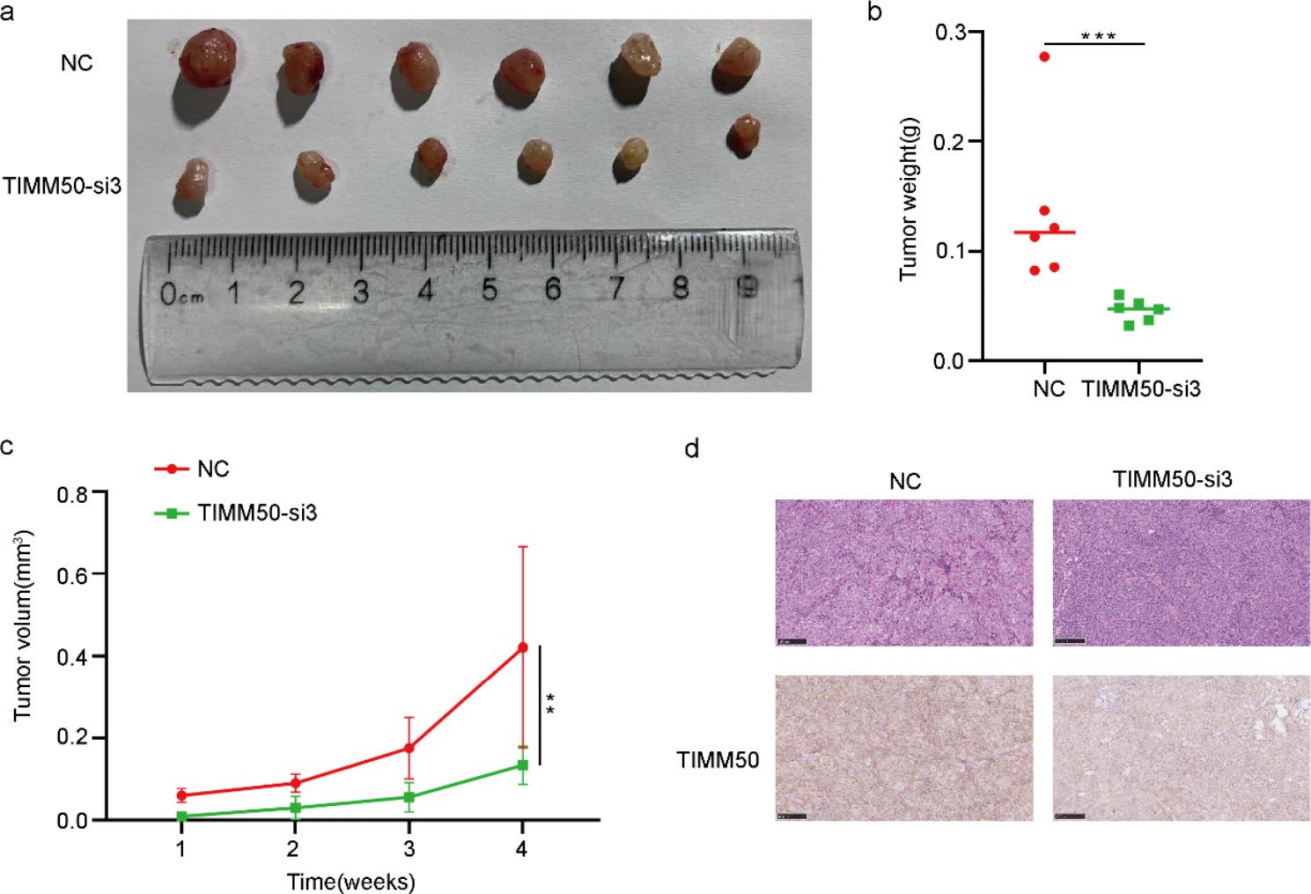
TIMM50 enhances tumour growth in vivo. **a** Images of subcutaneous injection of BALB/c nude mice. **b, c** Tumour volume and weight measurement in BALB/c nude mice. **d** Images of HE staining of subcutaneous xenograft tumours. The relative protein levels of TIMM50 were determined in subcutaneous xenograft tumours by IHC. Scale bar, 100 μm. n = 3, *P < 0.05, **P < 0.01, ***P < 0.001

To explore the effect of TIMM50 on the growth of EC cells in vivo, the animal experiments were performed. HEC-1B cells were stably transfected with TIMM50-sh3 and TIMM50-NC vectors were injected subcutaneously into female BALB/c nude mice, and tumor-related indices were measured once a week and removed after 4 weeks. The size and weight of tumors in the TIMM50-silenced vector group were significantly smaller than those in the NC group (Fig. 8a-c). HE staining (Fig. 8d) showed the cellular morphology of subcutaneously transplanted tumors. In addition, immunohistochemical staining of these subcutaneous tumors demonstrated that TIMM50 protein levels were significantly decreased in the TIMM50-sh3 group compared to the NC group (Fig. 8d). Overall, the results of these experiments revealed that TIMM50 could regulate the proliferation of EC cells in vivo.

## Discussion

Over the past 3 decades, the overall incidence of EC has increased by 132%. Although 67% of patients presenting with early disease are associated with 81% 5-year OS, the 5-year OS for stage IVA and IVB EC is only 17% and 15%, respectively [31]. Lactate, which is now considered as an important carbon source for cellular metabolism, is a signaling molecule in normal chronically inflamed tissues and in cancerous tissues. Lactate produced by cancer cells is further excreted into the extracellular space and has a critical role in promoting cancer progression [32]. An increasing number of studies have shown that proton-coupled lactate efflux from cancer or stromal cells plays a critical role in preserving the acidic phenotype and promoting tumor progression by regulating TME, involving cell invasion, angiogenesis, survival signaling, metastasis development and evasion of immune surveillance [33]. In some types of cancer, high level of lactate dehydrogenase status have been shown to be an independent biomarker for predicting response to immune checkpoint inhibitors therapy [34, 35, 36]. However, the lactic acid in EC is not fully understood. Therefore, it is important to comprehensively investigate the mechanism of lactate on EC and the prognosis.

In our study, we identified 122 differentially expressed LRGs between EC tissues and normal tissues. Seven of these genes were identified using LASSO and Cox regression analysis to construct a lactate-related prognostic signature. Mutation load was calculated and TP53 was found to be the most mutation-prone gene. EC patients were classified into high- and low-risk groups based on the median risk score. The high-risk group was strongly associated with poor prognosis of EC patients. Morever, a nomogram plots were constructed by integrating the risk score with clinical factors (age, tumor stage and grade), which can be a guide for individual treatment follow-up. TME is composed of extracellular matrix, tumor-associated fibroblasts, endothelial cells, epithelial cells, and immune cells, which are essential in the occurrence, development, invasion, metastasis, and treatment of EC [37]. Recent EC subclassifications have led to increasingly targeted therapeutic treatments based on disease biology. EC subtypes with high tumor mutational burden (e.g. POLE mutation/high mutation and microsatellite instability (MSI)) are highly immunogenic and exhibit more tumor-specific neoantigens, leading to increased CD3^+^ and CD8^+^ tumor-infiltrating lymphocytes and compensatory upregulation of immune checkpoints [38]. In the TME, tumor cells consume most of the nutrients and secrete excess lactate into the extracellular microenvironment, leading to acidosis, angiogenesis and immunosuppression. Lactate also regulates the metabolism of innate and adaptive immune cells by inhibiting the function of CD8^+^ T cells, natural killer (NK) cells, natural killer T (NKT) cells and dendritic cells. In contrast, lactate facilitates the maintenance of immunosuppressive functions of FOXP3^+^ regulatory T (Treg) cells in an acidic environment. Furthermore, lactate enhances M2 polarization in alternatively activated macrophages, promoting angiogenesis and tumorigenesis [39]. We next exploited the correlation between risk scores and previously reported pan-cancer immune subtypes. In the present study, EC patients with C1, C2 and C4 subtypes showed higher risk scores compared to C3 immune subtypes. The high-risk group showed increased dendritic cell activation, while the low-risk groups showed increased plasma cells and Treg cells. These results demonstrate the unique characteristics of the EC immune microenvironment and provide a useful addition to basic research. The pRRophetic algorithm was used to explore the association between EC patients based on risk scores and their response to Src protein kinase inhibitors. We calculated the IC50s for these drugs in both low- and high-risk groups and observed that patients in the low-risk group were significantly more sensitive to Src protein kinase inhibitors.

Based on multifactorial COX regression analysis, genes with pvalue < 0.05 were ACAT2, TIMM50, ALDOB and NDUFA6 in our current study. The acyl-coenzyme A (CoA): cholesterol acyltransferase 2 (ACAT2) promoted proliferation and metabolism in hepatocellular carcinoma, colorectal cancer, and breast cancer [40, 41]. Fructose-1,6-bisphosphate aldolase (ALDO) B, an enzyme involved in fructose metabolism, promotes fructose metabolism to fuel glycolysis, gluconeogenesis and the pentose phosphate pathway. Studies have shown that ALDOB-mediated fructose metabolism drives metabolic reprogramming of liver metastases from colon cancer; loss of ALDOB activates insulin receptor(IR) signaling primarily through releasing IR/ALDOB interaction to promote de novo lipogenesis and hepatocellular carcinoma [42, 43]. Ubiquinone oxidoreductase subunit A6(NDUFA6) is an accessory subunit of NADH. Several researches have reported that NDUFA6 was correlated with prognosis of multiple myeloma and was involved in mitochondrial fitness to promote proliferation in glioblastoma cells [44, 45]. The p-value of TIMM50 was to be the most significant by multifactorial COX regression analysis and was a potential marker of EC prognosis. TIMM50 (mitochondrial inner membrane translocase 50), which is also known as TIM50, is the receptor subunit that directs the translocation of precursor proteins from the outer mitochondrial membrane (TOM complex) to the inner mitochondrial membrane (TIM23 complex). The previous study demonstrated TIMM50 could promote tumor proliferation and invasion in NSCLC by enhancing the phosphorylation of its downstream ERK/P90RSK signaling pathway [46]; TIMM50 facilitated proliferation, migration and chemoresistance in breast cancer cells [47, 48]. Our results were consistent with the previous studies. In vivo, TIMM50 promoted EC cell proliferation, migration and lactate production; in vitro, silencing TIMM50 suppressed the growth of subcutaneous transplanted tumors in mice. The present study merely described that TIMM50 contributed to EC proliferation, migration and lactate formation, but the mechanism of effect was ambiguous and need to be further explored.

The present study has several merits. First, the present study is the first to systematically examine LMRGs in EC. We additionally performed experiments to validate TIMM50 as a novel regulator of genes related to lactate metabolism in vivo and in vitro. Second, our prognostic model of LMRGs constructed based on risk scores showed high accuracy and was valuable for clinical translation. Third, our constructed LMRGs could also guide clinicians to select appropriate drugs for treating EC by comparing patients’ sensitivity to common anticancer drugs in high- and low-risk populations. However, the limitation is that the current algorithm is based on cell lines, which needs to be further validated by preclinical studies.

## Conclusion

In conclusion, this study identified and constructed LMRGs for the first time in EC, which showed high diagnostic accuracy in predicting OS in EC patients and linked to immunity and drug sensitivity. TIMM50, which was identified as a novel molecule mediating lactate metabolism, promoted EC cell proliferation, migration and lactate metabolism in vitro and in vivo. Future studies are expected to investigate the potential regulatory mechanisms of LMRGs in terms of how they regulate lactate formation and metabolism. We hope that the utility of the constructed LMRGs will also be validated by future clinical studies.

## Electronic supplementary material

Below is the link to the electronic supplementary material.

Supplementary Material 1

Supplementary Material 2

Supplementary Material 3

## Data Availability

The raw data and materials supporting the conclusions of this article will be made available by Rui Shi, without undue reservation.

## Abbreviations

EC: Endometrial cancer
IHC: Immunohistochemistry
qRT-PCR: Quantitative real-time polymerase chain reaction
GAPDH: Glyceraldehyde 3-phosphate dehydrogenase
LMRGs: Lactate metabolism-related genes
CCK8: Cell counting kit-8
SDS: Sodium dodecyl sulfate
PVDF: Polyvinylidene difluoride
OS: Overall survival
PFS: Progression free survival
KM: Kaplan-Meier
FPKM: Fragments per kilobase transcript
PCA: Principal component analysis
Tregs: T cells regulatory
GO: Gene ontology
KEGG: Kyoto Encyclopedia of Genes and Genomes
