# Supplementary material for: Lactate metabolism-related genes to predict the clinical outcome and molecular characteristics of endometrial cancer
Source: BMC Cancer. 2023 May 31;23:491. doi: 10.1186/s12885-023-10934-y (PMC10230708; doi:10.1186/s12885-023-10934-y)
Supplement: Supplementary file 1 — Supplementary Material 1 [file 12885_2023_10934_MOESM1_ESM.pdf]

Supplement Figure 1

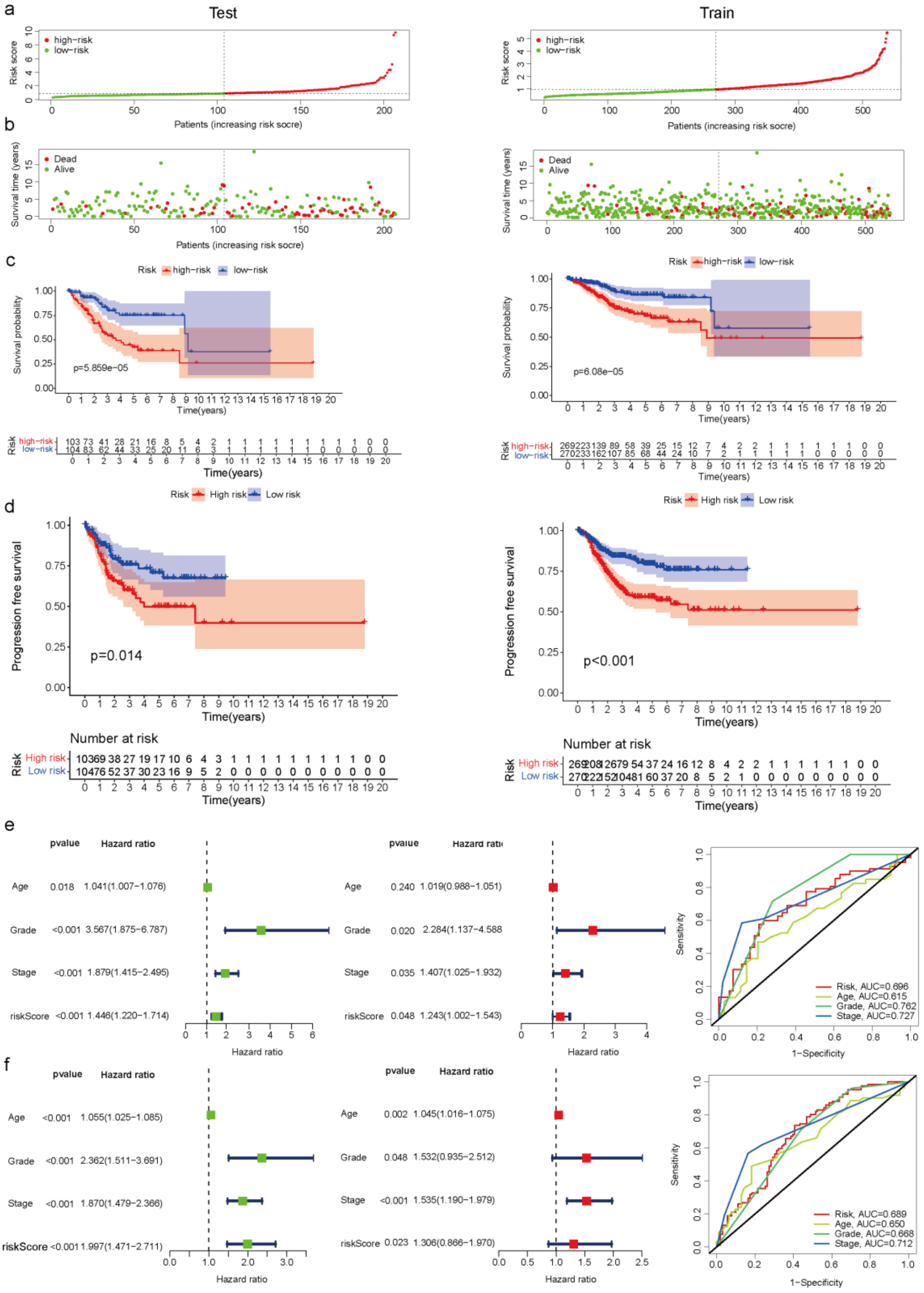

Prognosis value of LMRGs in the test and train set. a, b Risk score distribution and survival status of EC samples based on the risk score of the test and train sets. c, d The Kaplan–Meier survival curves of OS and PFS according to high-and low-risk groups in

patients with EC of the test and train sets. e The univariate Cox regression and multivariate analysis of age, grade, stage, and risk score for OS and ROC curve of the test set. f The univariate Cox regression and multivariate analysis of age, grade, stage, and risk score for OS and ROC curve of the train set.
