# Supplementary figures and images for: Lactate metabolism-related genes to predict the clinical outcome and molecular characteristics of endometrial cancer
Source: BMC Cancer. 2023 May 31;23:491. doi: 10.1186/s12885-023-10934-y (PMC10230708; doi:10.1186/s12885-023-10934-y)

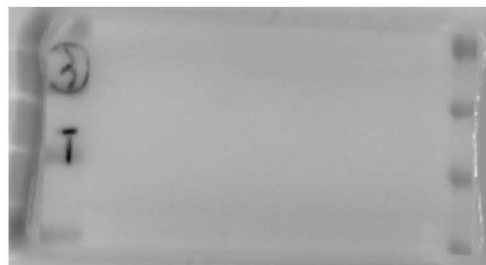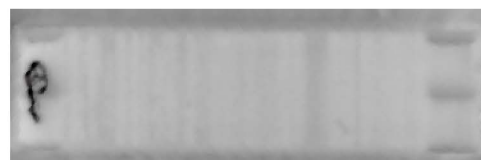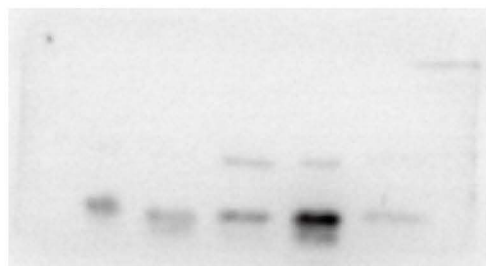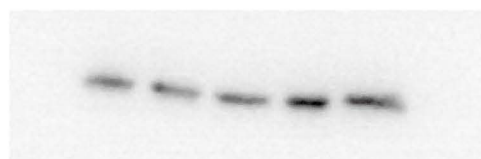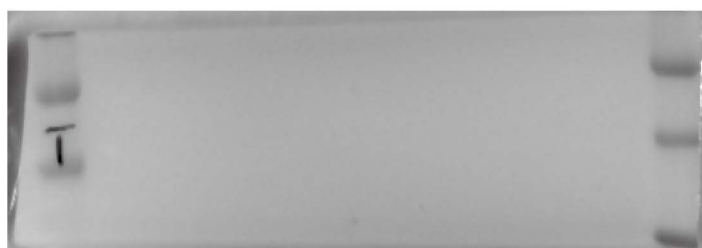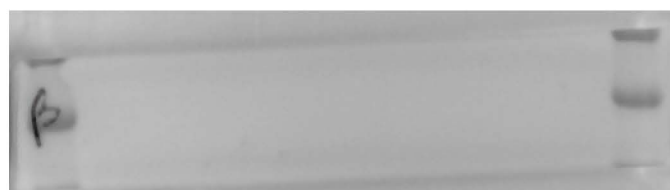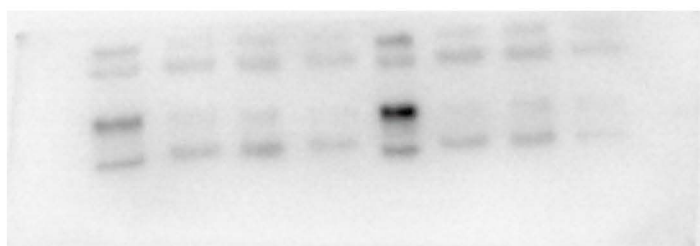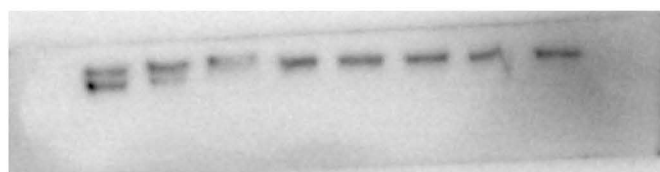

Supplement: Supplementary file 2 — Supplementary Material 2 [file 12885_2023_10934_MOESM2_ESM.pdf]

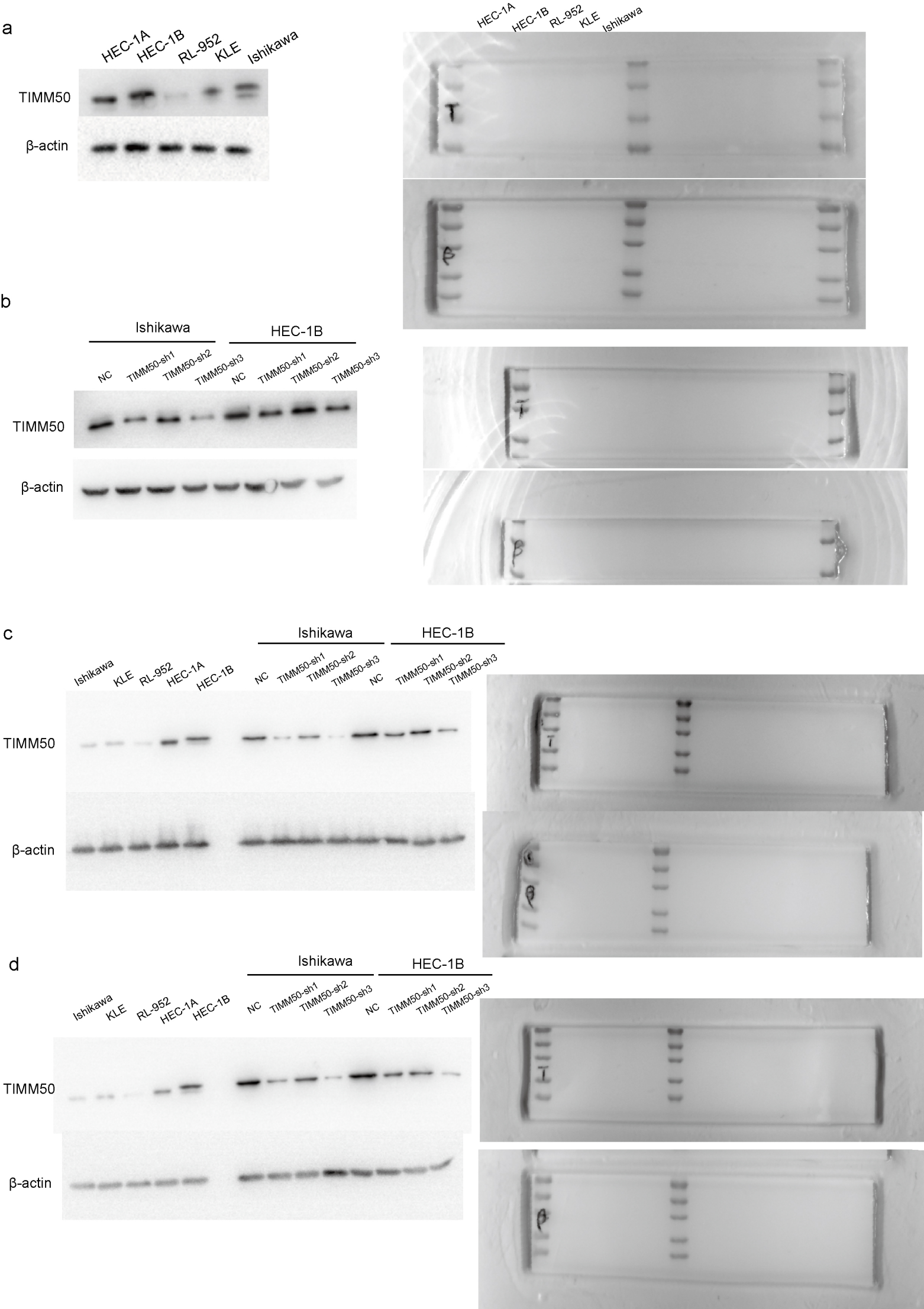

Supplement: Supplementary file 3 — Supplementary Material 3 [file 12885_2023_10934_MOESM3_ESM.pdf]
